# Fra-2 Is a Dominant Negative Regulator of Natural Killer Cell Development

**DOI:** 10.3389/fimmu.2022.909270

**Published:** 2022-06-22

**Authors:** Diana Schnoegl, Mathias Hochgerner, Dagmar Gotthardt, Leigh M. Marsh

**Affiliations:** ^1^ Ludwig Boltzmann Institute for Lung Vascular Research, Graz, Austria; ^2^ Institute of Pharmacology and Toxicology, University of Veterinary Medicine, Vienna, Austria; ^3^ Otto Loewi Research Center, Division of Physiology, Medical University of Graz, Graz, Austria

**Keywords:** Natural killer (NK) cell, differentiation, innate immunity, AP-1, activator protein 1, Fra-2

## Abstract

Natural killer (NK) cells play an important role in recognizing and killing pathogen-infected or malignant cells. Changes in their numbers or activation can contribute to several diseases and pathologies including systemic sclerosis (SSc), an autoimmune disease characterized by inflammation and tissue remodeling. In these patients, increased expression of the AP-1 transcription factor, Fra-2 was reported. In mice ectopic overexpression of Fra-2 (TG) leads to SSc with strong pulmonary fibrosis, pulmonary hypertension, and inflammation. Analysis of the underlying immune cell profile in the lungs of young TG mice, which do not yet show any signs of lung disease, revealed increased numbers of eosinophils and T cells but strongly reduced NK numbers. Therefore, we aimed to identify the cause of the absence of NK cells in the lungs of these mice and to determine the potential role of Fra-2 in NK development. Examination of inflammatory cell distribution in TG mice revealed similar NK deficiencies in the spleen, blood, and bone marrow. Deeper analysis of the WT and TG bone marrow revealed a potential NK cell developmental defect beginning at the preNKP stage. To determine whether this defect was cell-intrinsic or extrinsic, mixed bone marrow chimera and *in vitro* differentiation experiments were performed. Both experiments showed that the defect caused by Fra-2 was primarily cell-intrinsic and minimally dependent on the environment. Closer examination of surface markers and transcription factors required for NK development, revealed the expected receptor distribution but changes in transcription factor expression. We found a significant reduction in Nfil3, which is essential for the transition of common lymphoid cells to NK committed precursor cells and an AP-1 binding site in the promotor of this gene. In Summary, our data demonstrates that regulation of Fra-2 is essential for NK development and maturation, and suggests that the early NK dysfunction plays an important role in the pathogenesis of systemic sclerosis.

## Introduction

Natural killer (NK) cells represent 5-20% of circulating lymphocytes in humans and about 2-5% of lymphocytes in mice. Mature NK cells are found within the circulation but also present as tissue resident cells in various organs, including the lung, liver, uterus and gut, where they can recognize and kill infected or transformed cells (1). Changes in NK cell numbers or activation can contribute to several diseases including pulmonary hypertension (2), systemic sclerosis (3) or pulmonary fibrosis (4).

NK cells differ from innate lymphoid cells type 1 (ILC-1) in their surface receptor expression, cytokine responsiveness and function (5). Like other lymphoid cells, NK cells and ILC-1 develop in specialized niches in the bone marrow and are dependent on very similar transcriptional regulators (5). Both originate from multipotent, self-renewing hematopoietic stem cells, followed by the common lymphoid progenitors (CLPs) (1). NK cells then develop from the first NK cell committed precursor, the preNKP/pre-pro-NKP cells (6). Each stage in NK cell differentiation is characterized by the sequential up- and downregulation of surface receptors, including CD122 and CD127, which confer responsiveness to specific cytokines and growth factors e.g. IL-15 and IL-7 (6–9). Upon receptor binding IL-15 induces the expression of the Zinc finger transcription factor E4BP4/NFIL3, which is essential for the commitment to the NK lineage and transition from NKP to immature NK (iNK) (10). To date, the complex transcriptional regulation in specific steps during NK development and maturation is not completely understood.

The activator protein (AP)-1 family of transcriptional regulators controls cellular behaviour such as proliferation, differentiation, and transformation during both normal development and under disease conditions. They act as dimers and are activated within minutes in response to internal or external stimuli (11, 12). While the Jun family members (c-Jun, JunB and JunD) are able to homo-dimerise, the Fos proteins (c-Fos, Fra-1, Fra-2 and FosB) must form heterodimers with transcription factors of the Jun family. Fra-2 is one of the lesser-studied members of the AP-1 family but plays an important role in regulating deposition of extracellular matrix, mucus production and inflammation (12). Fra-2 often works in a positive feedback loop, where Fra-2 is induced by several inflammatory stimuli, which in turn activates the transcription of inflammatory mediators that feedback to activate Fra-2 (12). Fra-2 has been shown to play an important role in development and function of several immune cell types. In macrophages, Fra-2 drives their polarization towards alternatively activated, pro-fibrotic (M2) macrophages (13) and acts pro-inflammatory by impairing development and proliferation of regulatory T cells (14). In addition to its pro-inflammatory activity, Fra-2 has previously been shown to be important for the differentiation and proliferation of B cells and the expression of CCR4 and IL-5 on T cells (15, 16). Loss of Fra-2 leads to an increase in iNKT cells, due to an altered selection of iNKT cells in the earliest precursors. The authors suggest that normally Fra-2 would limit the amount of T cell precursors being able to enter the iNKT lineage (17). However, only little is known about the role of AP-1 transcription factors and especially Fra-2 in regulating NK cells.

In mice transgenic overexpression of Fra-2 (TG) leads to increased immune cell infiltration in several organs and autoimmunity (14, 18). These mice resemble a phenotype of human systemic sclerosis, with age-dependent remodelling of tissue and vasculature in several organs. This leads to strong fibrosis, mainly affecting the lungs and the skin, pulmonary hypertension and associated increased inflammatory cell recruitment (12, 14, 19). However, the mechanisms underlying the Fra-2-induced changes in the immune cells are still relatively unclear. In this study, we focused on the inflammatory changes in Fra-2 TG mice and have identified NK cells as one of the earliest affected cell populations. Furthermore, we determined that Fra-2 plays a vital role as negative regulator in the development and maturation of NK cells.

## Material and Methods

### Animal Models

Fra-2 transgenic (TG) mice, on a mixed BL6CBA background, were kindly provided by gift of Erwin Wagner and obtained from the Research Institute of Molecular Pathology, Vienna (19). Mice were back-crossed for least seven generations to a C57BL6/J background and *via* transgenic, wild-type crosses. TG and wild-type (WT) littermates were bred in-house under specific pathogen free conditions in isolated ventilated cages (IVC) with 12 hours light/dark cycles. Water and chow were supplied *ad libitum*. Mice aged between 6 and 14 weeks of both sexes were used in this study. All experiments confirmed to EU guidelines 2010/63/EU and were approved by the Austrian Federal Ministry of Science, Research and Economics (ethical permit number: 2020-0475.515). All measures were taken to keep animal suffering to a minimum.

### Bone Marrow Chimera Experiments

Six weeks old male Fra-2 TG mice and WT littermates were lethally irradiated with an irradiation dose of 8 grey in a Biological Irradiator Rad Source RS-2000. Donor bone marrow (BM) cells were isolated from 4-8 weeks old littermates and 5x10^6^ (mixed 50% WT and 50% TG donors) and injected into the tail vein. Six weeks after bone marrow transplantation, mice were sacrificed, and organs were harvested for analysis by flow cytometry and immunohistochemistry.

### Preparation of Single Cell Suspensions

Lung homogenates were prepared as previously described (20, 21). In brief, after perfusion one lobe was harvested, mechanically disassociated and enzymatically digested with 0.7 mg/ml Collagenase and 30 μg/ml DNAse in RPMI medium supplemented with 10% FCS, 2 mM glutamine and 1% penicillin-streptomycin (ThermoFisher Scientific) for 40 min at 37°C and then strained through a 100 µm cell strainer. Mononuclear cells were isolated from liver as above, followed by density gradient centrifugation using lymphospin medium (Pluriselect) and centrifuging for 15 minutes, 800 g. Bone marrow (BM) cells were isolated by flushing mouse femur and tibia and filtering disassociated bone marrow through a 100 µm cell strainer. Splenocytes were obtained by mechanically disassociating the spleen and straining it through a 100 µm cell strainer. Erythrocytes were removed from all single cell suspensions *via* ammonium chloride lysis.

### NK Cell Precursor Isolation and Differentiation

NK precursors were enriched from BM suspensions *via* magnetic depletion of CD3, B220, Gr-1, Ter119, CD11c and CD49b positive cells (antibody details are given in **Table 1**), using Dynabeads biotin binder magnetic beads (ThermoFisher Scientific). Isolated cells were seeded on mitomycinC (Stem Cell) deactivated OP9 feeder cells (ATCC) in MEMα medium, supplemented with 20% FCS, 1% penicillin-streptomycin 10 ng/ml FLT3L, 10 ng/ml IL-7 and 10 ng/ml IL-15 (all from Biolegend). NK cell differentiation was quantified by flow cytometry.

**Table 1:** Flow Cytometry Antibodies.

Antigen	Label	Dilution	Clone	Company	Figure
B220	bio	200	RA3-6B2	Biolegend	**3**, **5**
Caspase	APC	100	Rabbit	R&D Systems	**2**
CCR2	PE	50	SA203G11	Biolegend	**2**
CCR5	APC	50	HM-CCR5	Biolegend	**2**
CD11b	BV510	50	M1/70	eBioscience	**1**
CD11c	BV421	50	N418	Thermo Fisher	**1**
CD122	APC-Cy7	50	TM-b1	Biolegend	**5**
CD127	PE-CF594	20	A7R34	Biolegend	**5**
CD135	PE	50	A2F10	Biolegend	**5**
CD135	PE-Cy7	50	A2F10	Milteyni	**1**
CD19	APC-Cy7	100	6D5	biolegend	**1**, **2**, **4**
CD1d	APC	50	1B1	Biolegend	**3**
CD24	PerCP Cy5.5	500	M1/69	BD Biosciences	**1**
CD244.1	APC	50	C9.1	eBioscience	**5**
CD244.2	APC	100	m2B4	Biolegend	**5**
CD3	BV510	20	145-2C11	Sirigen	**1**, **2**, **3**, **4**
CD3	bio	100	145-2C11	Biolegend	**3**, **5**
CD4	APC	100	GK1.5	eBioscience	**1**, **2**, **5**
CD45	AF700	100	30-F11	BD Biosciences	**1**, **2**, **3**, **4**, **5**
CD49a	PE-Cy7	20	REA493	Milteyni	**3**
CD49b	bio	50	DX5	Biolegend	**4**
CD49b	PE-Cy7	20	DX5	Biolegend	**1**, **2**, **4**, **5**
CD49b	PE-CF594	20	DX5	Biolegend	**2**, **3**
CD64	APC	20	X-54-5/7.1	BD Biosciences	**1**
CD8	PE	100	53-6.7	Biolegend	**1**, **2**, **4**
CX3CR1	PE-Cy7	100	SA011F11	Biolegend	**2**
CXCR3	PerCP Cy5.5	10	CXCR3-173	eBioscience	**2**
Eomes	PE	10	REA116	Milteyni	**3**, **5**
Ets1	PE	100	C-4	Santa Cruz	**5**
GATA3	PE	10	REA174	Milteyni	**5**
Gr1	bio	1000	RB6-8C5	eBioscience	**3**, **5**
Gr-1	PE-Cy7	800	RB6-8C5	Biolegend	**1**
ID2	PE	50	ILCID2	Thermo Fisher	**5**
Ki67	APC-Cy7	100	Rat/SOLA15	eBioscience	**2**
MHC-II	APC-Cy7	400	M5/114.15.2	Biolegend	**1**
Nfil3	PE	100	S2M-E19	Thermo Fisher	**5**
NK1.1	SB600	20	PK136	eBioscience	**3**
NKp46	PerCP-Cy5.5	20	29A1.4	Biolegend	**1**, **2**,**3**, **4**, **5**
NKp46	SB600	10	29A1.4	Thermofisher	**2**
Streptavidin	BV421	400		Biolegend	**3**, **5**
Streptavidin	APC Cy7	1000		Biolegend	**3**, **5**
Sca1	PE-Cy7	20	D7	Thermo Fisher	**5**
Sca1	AF700	100	D7	Biolegend	**5**
Siglec F	PE	20	E50-2440	BD Pharmingen	**1**
T-bet	PE	50	REA102	Milteyni	**5**
Tcf1	PE	100	S33-966	BD Biosciences	**5**
TER119	bio	500	TER-119	eBioscience	**3**, **5**

### Flow Cytometry

Isolated cells were stained with Live/Dead Fixable Dead Cell Stain (ThermoFisher Scientific) and marker staining performed with panels of surface or intracellular marker antibodies (**Table 1**). Intracellular FACS staining was performed using the FoxP3 transcription factor buffer set (ThermoFisher Scientific). Whole blood samples were stained with surface antibodies, antibodies, afterwards erythrolysis was performed using FACS Lysing solution (BD Biosciences) according, to the manufacturer’s instructions. Samples were analysed with a Cytoflex LX or Cytoflex SI Flow Cytometer (Beckman Coulter) and evaluated using FlowJo (Biosciences, Ashland, OR, USA). For tSNE plots and histograms, CD45 positive cells were downsampled to equal numbers of events and samples concatenated (min 3 mice per group) to cluster the cells according to their marker and physical similarity.

### DNA Microarrays

In brief, RNA was isolated from the lungs of 8 week old Fra-2 TG and WT mice using the RNeasy Mini kit (Peqlab, Erlangen, Germany). The Low-input QuickAmp Kit (Agilent Technology, Santa Clara, CA) was used according to the recommended protocol, to pre-amplify and label the RNA with Cy5. Agilent 6x80K mouse microarrays were used for hybridization reactions in Agilent hybridization chambers 18 hours at 42°C. The microarray data was analysed with the Limma package in R. Intensity values were corrected for background signal quantile normalized. A list of chemokines, known for being responsible for NK recruitment to the lungs was used to filter genes of interest. Significance of differential expression was estimated using moderated t-statistics. Microarray data from Fra-2 TG and WT were in part previously published (22). Array data is available in NCBI’s Gene Expression Omnibus (GEO) Series *via* accession number GSE200425.

### Multicolour Immunofluorescence and Microscopy

For multi-colour immunofluorescence, 2.5 µm paraffin-embedded lung sections from 8 and 16 weeks old Fra-2 TG and WT mice were used. Slides were deparaffinised using ethanol and antigen retrieval was performed using AR6 Buffer (Perkin-Elmer) in an antigen retrieval chamber for 15 minutes at 200W. Slides were blocked with Perkin-Elmer Antibody Diluent/Block solution for 10 minutes at room temperature in humidified chamber and primary antibodies (**Table 2**) were diluted in Perkin-Elmer Antibody Diluent/Block solution. After washing with TBS-T (274 mM NaCl, 47.6 mM Tris HCl + 2% v/v Tween20 in H_2_O) HRP Opal Polymer 650 or Alexa-Fluor-conjugated secondary antibodies were incubated for 1 hour at room temperature. Nuclear counterstaining was performed with DAPI solution (ThermoFisher Scientific). For imaging a TCS-SP8 (DMi8 inverted microscope with a LIAchroic scan head) lightning confocal microscope (Leica), following a sequential workflow. The following objectives were used: Plan Fluotar 40x/1.25 glycerol immersion objective. Images were acquired at 2048 x 2048 and a pixel size of 142 x 142 nm.

**Table 2:** Antibodies for immunofluorescence staining.

Antibody	Dilution	Host	Company	Clone	Detection
SMA Cy3	1:750	Mouse	sigma	1A4	
Col1	1:500	goat	Southern Biotech	Polyclonal	AF488 donkey x goat 1:500
CD45	1:250	Rabbit	Abcam	Polyclonal	OPAL 650
DAPI	1:2000		Thermo scientific		

### *In Silico* Transcription Factor Binding Analysis

In silico analysis of transcription factor binding sites was performed using the ConTra v3 web server. Promoter regions of genes for natural killer cell development and function up to 1000 bp 5′ of the transcription start site were analysed with *mus musculus* as reference organism. For visualization, we used the positional weight matrix motif V$AP1_CM00199 (TRANSFAC20113 database) with a core stringency of 0.90 and similarity stringency of 0.75.

### Statistics

Data was assembled and analysed in R. For two groups data was compared using the Wilcoxon test and a p value <0.05 was considered statistically significant. Each data point represents one biological replicate with a line at median or an additional boxplot to show data range. For heatmap and principal component analysis, flow cytometry data was log_(10 + 1)_ transformed, hierarchical clustering was performed and the Euclidean distances plotted. To exclude experiment specific effects in transcription factor expression, mixed models were applied, with the mouse genotype and cell type as fixed factors and the individual mouse as a random factor (genotype*Cell_name, random = ~1|mouse_ID). NK differentiation data was analysed by mixed effects models with Bonferroni’s multiple comparisons test. Graphs were prepared using the R packages ggplot2 and pheatmap.

## Results

### Fra-2 Overexpression Causes a Systemic Decrease in NK Cells

**Figure 1:**
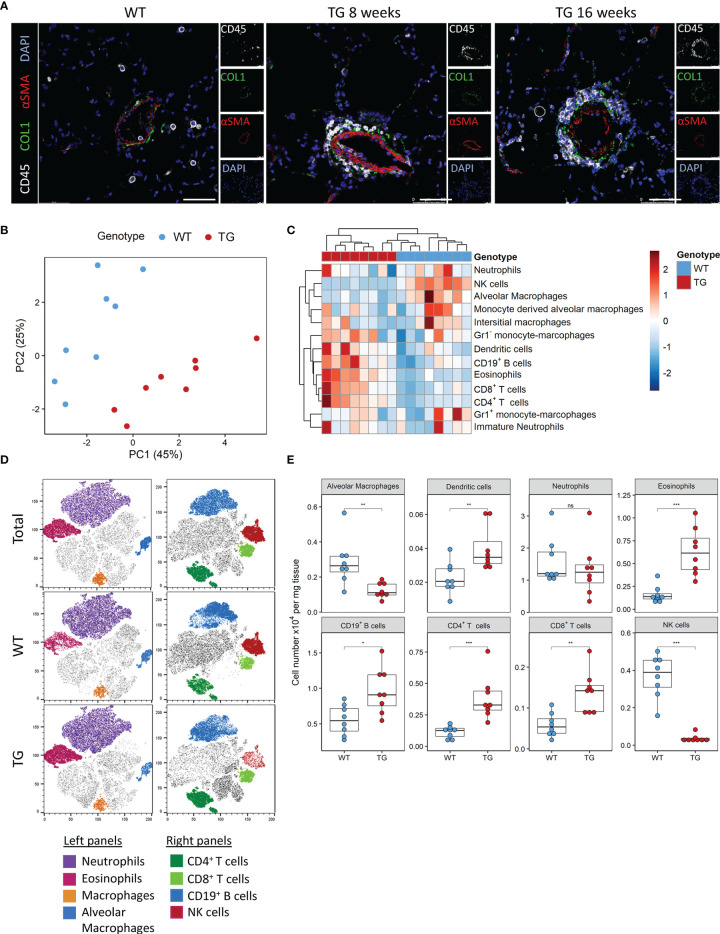
Fra-2 TG mice display an altered immune profile already before onset of disease. **(A)** Multicolour immunofluorescence of lung tissue of wild-type (WT) and Fra-2 TG mice, aged 8 and 16 weeks. White: CD45, Green: COL1, Red: aSMA, blue: DAPI. Scale bar 50µm. **(B)** Principal component analysis of the inflammatory cell profile in the lungs of 8 weeks old Fra-2 TG and WT mice as determined by flow cytometry. **(C)** Heatmap highlighting the changes in the abundance of immune cells. **(D)** tSNE plots of concatenated CD45^+^ immune cells; overlayed are populations of myeloid (left) and lymphoid (right) immune cells. **(E)** Significantly altered populations of immune cells in lung tissue. Statistical differences were determined with a Wilcoxon Rank Sum test, ^ns^p>0.05, *p<0.05, **p<0.01, ***p<0.001, n=8.

We previously observed that ectopic overexpression of the transcription factor Fra-2 results in pronounced inflammation and age-dependent remodelling of the lungs and other organs. We now performed a closer examination of the underlying inflammation in the lungs at 8 and 16 weeks, time points where lung remodelling is not yet apparent (8 weeks) or first developing (16 weeks) (21, 22). Multi-colour immunofluorescence staining for α-smooth muscle actin (red), collagen 1 (green) and CD45 (white) revealed perivascular and peribrochial inflammation at both timepoints, at 16 weeks inflammatory cells (CD45^+^) were also increasingly present in the lung tissue (**Figure 1A**).

To understand these early inflammatory changes in more detail, we performed comprehensive immune profiling of the lungs isolated from 8 week old Fra-2 TG and WT mice by flow cytometry. A representative gating strategy is shown in **Supplementary Figure 1A**. Already at this young age, the altered inflammatory profile in Fra-2 TG mice was clearly visible when compared to aged matched control mice, with samples strongly separating in principal component analysis (**Figure 1B**). Hierarchical clustering clearly separated the WT and TG mice, with specific cell populations being more abundant in TG mice (**Figure 1C**). Consistent with previous studies (21, 23), we found significantly increased eosinophils, CD19^+^ B cells, CD4^+^ and CD8^+^ T cells but reduced amounts of alveolar macrophages, (**Figures 1C, D**). Visualisation of the flow cytometry data using tSNE (t stochastic neighbourhood embedding) plots, which cluster cells according to their marker and physical similarity, exemplified these differences (**Figure 1D**). Interestingly despite a strong increase in almost all lymphoid cell populations in the lungs of TG mice, NK cells were almost completely absent (**Figures 1B–E**). Due to this unexpected finding, we investigated the underlying causes of this NK deficiency.

### Reduced Recruitment, Proliferation, or Survival Does Not Fully Account for the Lung NK Cell Deficiency

**Figure 2:**
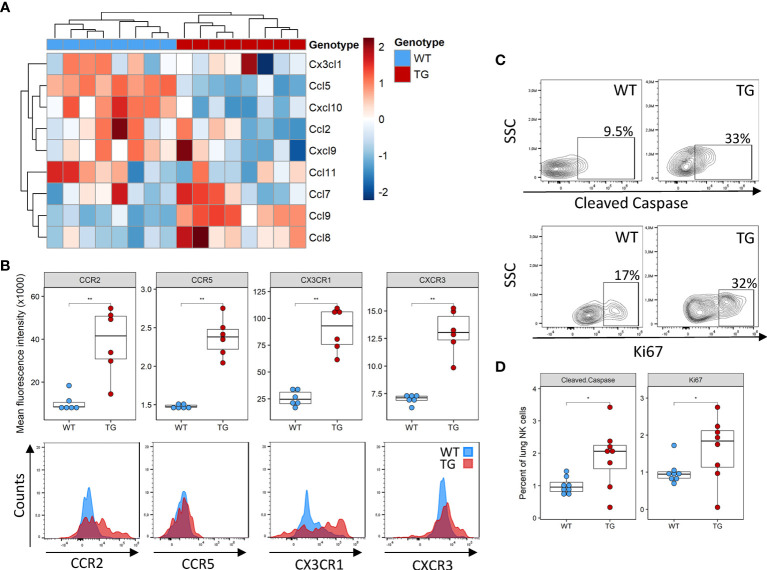
Altered recruitment factor expression and NK cell turnover in the lungs of Fra-2 TG mice **(A)** Heatmap of mRNA levels of NK recruiting chemokines in the lungs of 8 week Fra2-TG and WT mice. **(B)** Flow cytometry analysis of NK cell chemokine receptors expression. **(C)** Representative plots of intracellular flow cytometry staining of Cleaved Caspase and the proliferation marker Ki67. **(D)** Respective quantification. Statistical differences were determined with a Wilcoxon Rank Sum test, *p<0.05, **p<0.01, n=8.

Several potential mechanisms may potentially explain the lower NK cell number in the lung, including decreased recruitment, decreased local proliferation or increased apoptosis. NK cells are recruited to the tissue in response to a wide array of chemokines, such as Ccl2, Ccl3, Ccl5, Ccl7, Ccl8, Ccl9, Ccl11, Ccl13, Cxcl9‐11 and Cx3cl1 (24). We examined their expression in the lung tissue using existing DNA microarrays (22). Compared to WT mice, Fra-2 TG mice exhibited elevated levels of Ccl8 and Ccl9, but lower expression of Ccl5 and Cxcl10 (**Figure 2A** and **Supplementary Figure 2A**). We next used flow cytometry to quantify the expression levels of the corresponding chemokine receptors CCR5, CCR2 and CX3CR1 on NK cells and found increased expression of all these receptors on transgenic NK cells, possibly a compensatory mechanism (**Figure 2B**). Due to this mixed expression of responsible cytokines and receptors, we hypothesized that recruitment alone does not lead to the observed absence of NK cells in TG lungs.

Next, we examined local proliferation and apoptosis of NK cells in the lungs by intracellular staining for the proliferation marker Ki67 and the apoptosis regulator cleaved caspase. Overall levels of homeostatic NK cell proliferation and apoptosis were low in both WT and Fra-2 TG mice with a higher NK cell proliferation and apoptosis in the lungs of Fra-2 TG mice (**Figures 2C, D**). This potentially indicates increased turnover, however, it is unlikely to completely explain the lack of NK cells in the lungs of TG mice. Altogether, these observations suggest that the decrease of NK cells in Fra-2 TG mice is not solely caused by a local deficiency, but may arise from a more systemic fault.

### Fra-2 TG Mice Display Reduced Numbers of NK Cells in All Tissues

**Figure 3:**
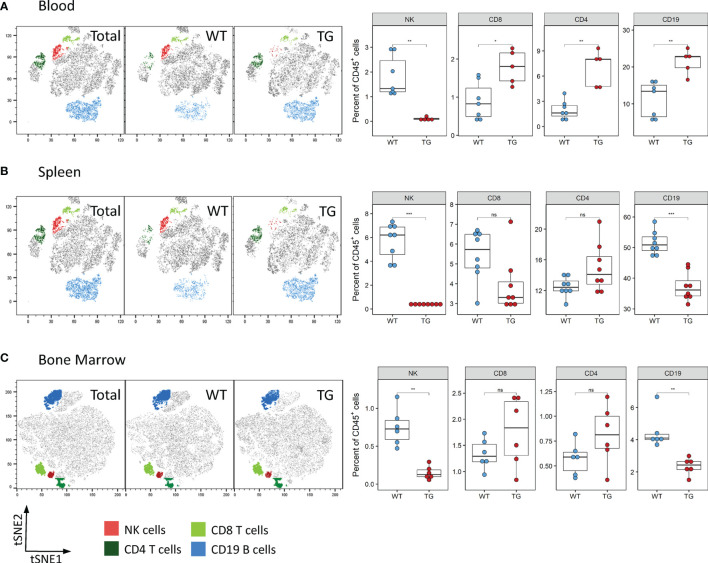
Fra-2 TG mice display reduced numbers of NK cells in all tissues. Flow cytometry analysis of lymphocytes in the blood **(A)**, spleen **(B)** and bone marrow **(C)** of wild-type (WT) and Fra-2 TG mice. tSNE plots of concatenated CD45+ from the respective tissue with overlaid gated cell populations. Statistical differences were determined with a Wilcoxon Rank Sum test, ^ns^p>0.05 *p<0.05, **p<0.01, ***p<0.001, n≥5.

We next determined whether the observed reduction of NK cells was also visible in other organs. Analysis of the blood and spleen also revealed significantly decreased absolute and relative numbers of NK cells in circulation and splenic NK cells in Fra-2 TG mice (**Figures 3A, B** and **Supplementary Figure 3**). As NK cells develop in the bone marrow before being released to the circulation, we next analysed NK cells in this compartment. Within the CD45^+^ population, a decreased proportion of mature NK cells was observed, while numbers of CD4^+^ and CD8^+^ T cells were unchanged (**Figure 3C**). Interestingly, amounts of CD19^+^ B cells were increased in the lungs and circulation but decreased in spleen and bone marrow. Taken together, this suggests that Fra-2 overexpression potentially causes a defect in NK cell development, leading to a systemic absence of NK cells in TG mice.

### Fra-2 Overexpression Causes a Cell-Intrinsic Defect in NK Development

**Figure 4:**
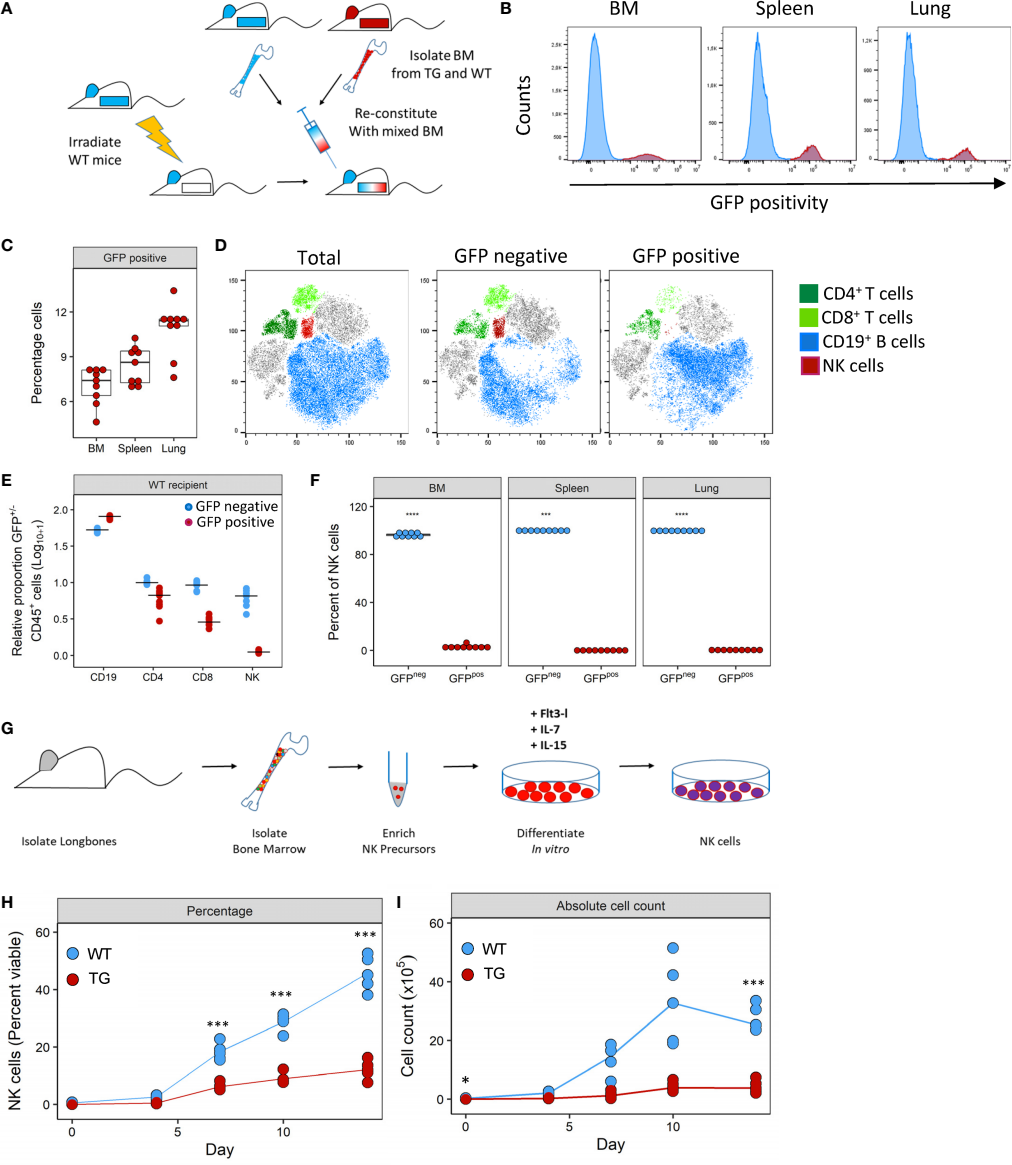
Fra-2 overexpression leads to a cell-intrinsic defect in NK development. **(A)** Schematic representation of mixed bone marrow (BM) chimera experiment. BM was extracted from WT and Fra-2 TG mice, mixed 1:1 and injected into irradiated WT mice, and mice analysed six weeks post transfer. **(B)** Histograms of the GFP signal in total immune cells in different compartments of WT mice post BM reconstitution; y-axis represents percentage viable counts in the bone marrow (BM), and percentage CD45^+^ counts in the spleen and lung and **(C)** their quantification. **(D)** tSNE plots of concatenated CD45^+^ cells in the lung and overlaid lymphocyte populations split according to GFP positivity (negative = cells of WT origin and positive = TG origin). **(E)** Relative proportion of CD45^+^/GFP^-^ (blue) and CD45^+^/GFP^+^ (red) cells in the lungs of WT recipient mice, line shows median. Data was log_10+1_ transformed to allow all cells to presented on the same axis. **(F)** Quantification of NK cell GFP positivity in the BM, spleen and lungs of WT mice. **(G)** schematic overview of the NK cell *in vitro* differentiation experiments from bone marrow precursors. NK precursors were enriched by magnetic depletion of lineage positive cells (CD3, B220, Gr1, Ter119, CD11c and CD49b positive cells) and stimulated with FLT3L, IL-7 and IL-15 to stimulate NK cell differentiation. Amount of *in vitro* generated NK cells over time, shown as percentage viable **(G)** or absolute cell count **(H)**. Statistical differences in **(C, F)** were determined with a Wilcoxon Rank Sum test, and **(H, I)** using mixed effects models with Bonferroni’s multiple comparisons test. *p<0.05, ***p<0.001, ****p<0.0001, n≥5.

To investigate how Fra-2 overexpression may potentially interfere with NK cell development, we performed mixed bone marrow chimera experiments with an aim to determine the relative contribution of cell intrinsic and extrinsic effects of Fra2 overexpression on NK cell development (experimental schemes in **Figure 4A** and **Supplementary Figure 4A**). Six weeks post irradiation and transplantation with mixed WT and TG bone marrow, inflammatory cells in the bone marrow (BM), spleen and lungs were analysed. The presence of a GFP reporter gene in all TG cells enabled separation of both donor genotypes. After reconstitution, both GFP positive and negative CD45^+^ cells were detected in BM, spleen and lungs of WT mice. This is demonstrating that precursor cells derived from both WT and Fra-2 TG BM, give rise to inflammatory cells in both recipient genotypes. Of note, in both WT and TG mice the majority of cells in the BM was GFP negative, indicating a better engraftment of WT BM cells (**Figures 4B, C** and **Supplementary Figures 4D, E**). Analysis of the relative proportions of lymphocytes derived from WT or TG precursors (GFP negative or positive, respectively) in WT recipients revealed slight reductions in GFP^+^ CD4^+^ and CD8^+^ T cells and an increase in CD19^+^ B cells (**Figures 4D, E**). In contrast GFP^+^ NK cells were strongly absent in WT recipients (**Figures 4D, E**). Similar results were observed in TG recipients, with a strong lack of GFP^+^ NK cells, reduced CD8^+^ cells, but increased GFP^+^ CD4^+^ T cells and decreased GFP^+^ B cells (**Supplementary Figures 4D, E**).

Closer examination of NK cells in all compartments revealed that the vast majority of NK cells were GFP^-^ with 99.8% and 97.9% of NK cells in WT and Fra-2 TG recipients being GFP^-^ (**Figure 4F** and **Supplementary Figure 4F**). Therefore, almost all NK cells developed from WT precursors indicating a cell-intrinsic developmental defect. We next examined whether this *in vivo* developmental effect could be recapitulated *in vitro*. To investigate if Fra-2 TG precursor cells could be stimulated to differentiate into NK cells *in vitro*. Lineage negative NK precursors were isolated from the BM of Fra-2 TG and WT mice and differentiated *in vitro* (experimental scheme: **Figure 4G**; representative gating strategy: **Supplementary Figure 4**). In line with our *in vivo* findings, WT precursors gave rise to high numbers of NK cells, while TG precursors exhibited a much lower differentiation capability (**Figures 4H, I**).

### The Defect Caused by Fra-2 Affects All NK Committed Precursors

**Figure 5:**
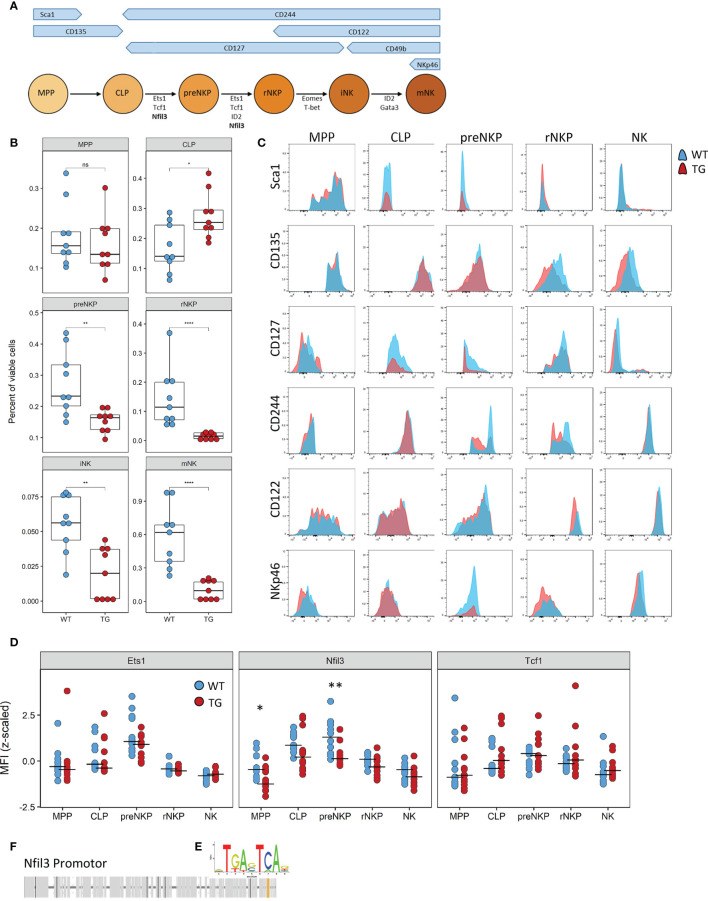
The defect caused by Fra-2 affects all NK committed precursors. **(A)** Schematic representation of surface marker expression and essential transcription factors during NK cell development in the bone marrow. **(B–D)** Flow cytometry analysis of NK cells in bone marrow of WT and Fra-2 TG mice. **(B)** Quantification of NK precursors in the bone marrow. **(C)** Surface Marker expression on NK precursors. **(D)** Quantification of NFIL3, Ets1 and Tcf1 on NK precursors *via* flow cytometry; Z-scored mean fluorescent intensity are shown, with a line at the median. **(E)** AP-1 consensus sequence. **(F)** In silico transcription factor binding site analysis for AP-1 in the promotor of Nfil3. Statistical differences were determined in **(B)** with a Wilcoxon Rank Sum test and in **(D)** with mixed models using mouse genotype and cell type as fixed factors and the individual mouse as a random factor, ^ns^p>0.05 *p<0.05, **p<0.01, ****p<0.0001.

NK cell differentiation is a highly structured process with cells developing along set trajectories. We therefore sought to determine at which stage Fra-2 overexpression interferes with the normal differentiation process. As each developmental stage is characterized by changes in surface marker expression (**Figure 5A**), we used this to identify and quantify the abundance of known NK precursors in the BM (A representative gating strategy is shown in **Supplementary Figure 5**). Equivalent proportions of the multipotent progenitor cells (MPP) and increased common lymphoid progenitor cells (CLP) were observed in Fra-2 TG mice. However, all NK-committed precursors, starting from the preNKP were significantly decreased (**Figure 5B**). The increased amounts of CLPs but strongly reduced numbers of all following precursors suggest a block during lineage commitment and transition from CLP to preNKP. This developmental defect may potentially be due to decreased receptor expression, which in turn would reduce the response to the growth factors required for differentiation. All NK precursors, in both WT and TG mice, displayed expected distribution of surface receptors, with high levels of Sca-1, CD135 and CD127 on the early precursors, a shift from CD127 to CD122 in the stage of rNKP and upregulation of CD244, CD122 and NKp46 in immature and mature NK cells (**Figure 5C**).

In addition to receptor expression, each differentiation stage requires the coordinated expression of several transcription factors (**Figure 5A**) Analysis of the key transcription factors required for the differentiation from CLP to iNK by flow cytometry, revealed a significant decrease in the Nfil3 (E4BP4) transcription factor, which is required in CLP, for commitment to the ILC-1 and NK lineages (**Figure 5D**). Other transcription factors, such as Ets1, Tcf1, Gata3, ID2, Eomes and T-bet, were unchanged (**Figure 5D** and **Supplementary Figures 6A, B**). In silico transcription factor binding site analysis reveals a potential AP-1 binding site, with the consensus sequence TGA-C/G-TCA (**Figure 5E**) in the promoter region of this gene (**Figure 5F**), suggesting the involvement of the AP-1 family in the regulation of Nfil3.

As NK-related cell types, such as ILC-1, share common progenitor cells and dependence on Nfil3 during their development, we assessed whether this NK-related cell type was also affected by Fra-2 overexpression. Analysis of ILC-1 in the lungs and liver by flow cytometry (gating strategy shown in **Supplementary Figure 8A**) revealed a significant decrease in ILC-1 and confirmed the absence of the NK cells in Fra-2 TG mice (**Supplementary Figure 8B**).

## Discussion

In this study we have shown that Fra-2 is a powerful negative regulator of NK cell differentiation, with overexpression of Fra-2 resulting in strongly reduced systemic NK cell numbers arising from a CLP to preNKP differentiation defect. In mice, ectopic overexpression of Fra-2 leads to systemic inflammation, with immune infiltrates in multiple organs (14, 18). Fra-2 has been shown to play a role in regulation and differentiation of several immune cell types and involvement of other AP-1 transcription factors have been reported to play important roles in regulating NK function, cytotoxicity and surface receptor expression (1, 25, 26). Our data expands on these studies and reveals the importance on Fra-2 in preventing the normal differentiation of NK cells.

Although, several studies have investigated the immune profile in Fra-2 TG mice, none investigated the presence of NK cells (13, 18, 21–23, 27). Consistent with previous studies we found increased numbers of eosinophils and T and B cells, but decreased alveolar macrophages (13, 21, 23). The decreased B cell abundance in the BM, but increased circulating and lung numbers, supports the known role of Fra-2 in B cell development and turnover (15).

Our study is the first to report almost complete absence of NK cells in the lungs of these mice. Several potential explanations could have explained this deficit. Local recruitment is a result of the coordinate action of chemokine – receptor interaction. Here we observed increased expression of Ccl7, Ccl8 and Ccl9 in the lung tissue, but a reduction of Ccl5 and Cxcl10. Due to this mixed expression, we postulated that this on its own, changes in recruitment factors would not be sufficient to explain the absence of NK cells in Fra-2 TG lungs. Interestingly, the remaining transgenic NK cells in TG generally possessed higher expression of NK recruitment receptors, which may represent a compensatory mechanism to the low NK numbers or a direct consequence of Fra-2 overexpression. The lungs of Fra-2 TG mice may be subjected to increased NK cell turnover as shown by the increased apoptosis and proliferation in TG NK cells, but again is unlikely to be the sole cause for the strong reduction in NK cell number. We therefore also analysed peripheral tissues, including the bone marrow, liver, lung, spleen, and peripheral blood, and showed almost a complete ablation of NK in all these organs. Due to this systemic lack of NK cells, we hypothesized that Fra-2 might cause a NK cell developmental defect. One alternative possibility, which cannot be completely excluded, is that the random insertion of the Fra-2 TG may have disrupted the mouse NK locus on chromosome six. However, several pieces of evidence argue against this possibility: 1) Due to the heterozygous TG/WT breeding strategy, all TG offspring would have one intact NK locus derived from the WT parent. 2) Expression data revealed that several genes within the NK cluster have decreased expression, while others are unregulated or possess increased levels (data not shown). 3) NK1.1 encoded by the Klrb1 (Killer cell lectin-like receptor subfamily B member 1) was expressed on the remaining NK and ILC-1 cells in TG mice (**Supplementary Figure 8**).

Based on our mixed bone marrow chimera experiments we identified a cell intrinsic developmental defect. Despite superior WT BM cell engraftment, both WT and TG derived lymphocytes were detected in the BM, lung, liver, blood and spleen of both recipient genotypes. The reasons for the low engraftment efficiency of Fra-2 TG BM is unclear, it may be due to lower fitness due to the transgene expression or potentially, but less likely, an immunogenic response against GFP reporter in TG cells (28). A stem cell defect or reduced repopulation capacity would be a possible explanation, but this question is beyond the scope of this manuscript and could be addressed in further studies. Finally, one conceivable is option that the GFP reporter gene present on the TG construct may be supressed or the GFP signal lost, which would decrease our capability to identify TG derived cells. Despite these potential limitations, we could conclusively show that NK cells in both recipient genotypes originated exclusively from the WT BM, while other CD45^+^ immune cells originated from both WT and Fra-2 TG precursor cells. Interestingly, TG recipients possessed high proportion of GFP^+^ CD4^+^ compared to GFP^-^ CD4^+^ T cells in their lungs, indicating a potential selective advantage of these cells in a transgenic environment and is consistent with the high abundance of CD4^+^ cells in the lungs of non-chimeric TG mice (21, 22). In line, TG recipients of mixed BM show a decreased relative abundance in other GFP^+^ lymphoid cell populations.

NK cell differentiation is a highly coordinated process characterized by the sequential expression of several receptors, which confer cytokine specificity. Receptor binding subsequently activates intracellular signalling cascades and transcription factors. Similar to B and T cells, NK cells originate from MPP and develop *via* CLP to the different lineages. The first precursors committed to the NK cell line are the preNKP/pre-pro-NKP cells (6, 7). In contrast to CLPs, NKP defined lineages do not express Sca1 and CD135, but express the markers CD244, CD117 and CD127. During development from preNKP to rNKP, CD127 is downregulated and CD122 is upregulated, indicating a shift from IL-7 to IL-15 dependence (8). As NK precursors in Fra-2 TG mice possessed similar receptor repertoire as WT precursors, we were able to exclude that the differentiation defect was due to changes in receptor expression.

Corresponding to the impaired differentiation from CLP to preNKP, we observed decreased expression of the essential differentiation factor Nfil3. As Nfil3 is required for correct development of both NK cells and ILC-1 (29, 30), differentiation of both cell types, should be, and was indeed impaired. Follow up experiments including limiting dilution assays and culture of specific precursors may give further insights in the CLP-preNKP differentiation block. Nfil3 has been shown to be essential for NK cell development and maturation by inducing expression of the transcription factors ID2 and GATA3, which are crucial for the late stage NK cell development from immature (iNK) to mature NK cells (mNKs) (10, 31, 32). These findings are slightly controversial as certain NK subsets, such as TRAIL^+^ liver NK cells, can develop in the absence of Nfil3, which might explain the origin of the remaining NK cells in the Fra-2 TG mice. As Nfil3 activity is only essential during lineage commitment and early developmental stages, when early development checkpoints are passed, Nfil3 is not required for NK cell maintenance and survival in the periphery (30, 33).

Nfil3 can be regulated by various factors, including posttranslational modifications, such as sumoylation and phosphorylation, or different cytokines and growth factors (34, 35). Nfil3 can be inhibited by TGF-β (36) or upregulated by IL-7 and IL-15. Both, IL-7 and IL-15 lead to the activation of JAK3/STAT5 signalling (37), which makes STAT5 a key regulator in NK development, maturation, survival and function (38). Fra-2 has been shown to be a STAT5 target gene, which is activated in response to IL-2 in human CD4^+^ T cells (39), possibly leading to an altered response to these cytokines in Fra-2 TG mice. Since the defect, caused by Fra-2, occurs in an early developmental stage, before the upregulation of CD122, in this case IL-7 is the more important activator of STAT5 signalling. Furthermore, Notch signalling, which is essential for the CLP-preNKP transition has been shown to activate Fra-2 (40). Together with the AP-1 binding site in the promotor of Nfil3, identified in our *in silico* transcription factor binding analysis, this suggests that the AP-1 transcription factor family and especially Fra-2 play an important role in regulating the commitment of CLP to the NK and ILC lineages. However, functional experiments are still required to investigate this interaction in more detail. Fra-2 possesses differential transactivation activity: in a dimer with c-Jun, Fra-2 lowers the transactivation activity compared to c-Jun/c-Fos, however upon dimerization with JunD, it increases the transactivation activity, compared to JunD homodimers (12), therefore overexpression of Fra-2 could not only influence target gene expression by direct binding but could also affect expression of Nfil3 by changing the composition of the AP-1 dimer that binds to its promotor.

In conclusion, we have shown that Fra-2 is a strong negative regulator of NK and ILC-1 development and acts *via* downregulation of the essential transcription factor Nfil3. Further studies could address questions of the effects of Fra-2 overexpression in mature NK cells on their function and the loss of Fra-2 in NK cells and their precursors.

## Data Availability Statement

The original contributions presented in the study are publicly available. This data can be found here: https://www.ncbi.nlm.nih.gov/geo/GSE200425.

## Ethics Statement

The animal study was reviewed and approved by Austrian Federal Ministry of Science, Research and Economics.

## Author Contributions

DS, DG, LM conceived and designed the study. DS, MH performed data acquisition and analysis. DS, LM wrote the manuscript. All authors contributed to the interpretation of the data and drafting manuscript. All authors read and approved the final version of the manuscript. All authors contributed to the article and approved the submitted version.

## Funding

DS was funded by the Austrian Research Promotion Agency (FFG, project number 870904), awarded to LM and was part of the MolMed PhD program of the medical university of Graz. DNA microarray analysis was supported *via* grants CRC1021 Z1 and CRU309 Z1 from the German Research Foundation to Jochen Wilhelm.

## Conflict of Interest

The authors declare that the research was conducted in the absence of any commercial or financial relationships that could be construed as a potential conflict of interest.

## Publisher’s Note

All claims expressed in this article are solely those of the authors and do not necessarily represent those of their affiliated organizations, or those of the publisher, the editors and the reviewers. Any product that may be evaluated in this article, or claim that may be made by its manufacturer, is not guaranteed or endorsed by the publisher.

## Supplementary Material

Supplementary Figure 1 Representative flow cytometry gating strategy of lymphoid and myeloid cell populations in the lungs. Lymphoid cells were gated on CD45^+^, side scatter low and B cells (CD19^+^), T cells (CD3^+^ followed by CD4/CD8/gdTCR) and NK cells (NKp46^+^ CD49b^+^). Myeloid cells were gated from CD45^+^ and further defined as alveolar macrophages (CD11c^+^, SiglecF^+^), neutrophils/PMN (CD11b^+^ Gr1^+^), Eos (SiglecF^+^), Monocytes (CD11b^+^, MCHII^+^, different populations based on their expression of CD64 and Gr1), Dendritic cells (CD11b^+^, MHCII^+^, CD24^+^), interstitial macrophages (CD11b+, MHCII^+^, CD64^+^, CD11c^-^), monocytes-macrophages (CD11b^+^, MHCII^+^, CD64^+^, CD11c^+^). Dotplots for both wild-type and Fra-2 transgenic mice are shown, with proportion of NK cells from the CD3^-^CD19^-^ gate.

Supplementary Figure 2 Expression levels of cytokines required for NK recruitment. Extracted cytokine expression values in the lungs of WT and Fra-2 TG mice using DNA microarrays. Statistical differences were determined with a Wilcoxon Rank Sum test, ^ns^p>0.05, **p<0.01, ***p<0.001, n = 8.

Supplementary Figure 3 Quantification of NK cell Numbers in blood and spleen. Absolute numbers of NK cells in the blood **(A)** and spleen **(B)** as determined by flow cytometry. Statistical differences were determined with a Wilcoxon Rank Sum test, ^ns^p>0.05, *p<0.05, **p<0.01, n = 5-7.

Supplementary Figure 4 Lymphoid cell differentiation effects in TG recipient mixed BM chimeras. **(A)** Schematic representation of mixed bone marrow (BM) chimera experiment. BM was extracted from WT and Fra-2 TG mice, mixed 1:1 and injected into irradiated Fra-2 TG mice. Mice were analysed six weeks post transfer. **(B)** Histograms of the GFP signal in total immune cells in different compartments of Fra-2 TG mice post BM reconstitution; y-axis represents percentage viable counts in the bone marrow (BM), and percentage CD45^+^ counts in the spleen and lung. **(C)** Quantification of GFP positive cells as shown in **(B)**. **(D)** tSNE plots of concatenated CD45^+^ cells in the lung and overlaid lymphocyte populations split according to GFP positivity (negative = cells of WT origin and positive = TG origin). **(E)** Relative proportion of CD45^+^/GFP^+^ and CD45^+^/GFP^-^ cells in the lungs of TG recipient mice. Data was log_(log10+1)_ transformed to allow all cells to presented on the same axis. **(F)** Quantification of NK cell GFP positivity in the BM, spleen and lungs. Statistical differences in F were determined with a Wilcoxon Rank Sum test, *p<0.05.

Supplementary Figure 5 Gating strategy for *in vitro* differentiation of murine NK cells. NK precursors were enriched by magnetic depletion of lineage positive cells (CD3, B220, Gr1, Ter119, CD11c and CD49b positive cells) and stimulated with FLT3L, IL-7 and IL-15 to stimulate NK cell differentiation. NK cells were defined as CD3^-^, CD49b^+^, NKp46^+^ cells.

Supplementary Figure 6 Gating strategy for identifying and analysing NK cell precursors in the bone marrow. Bone marrow were analysed by flow cytometry using two antibody panels identify cells of the MPP- rNKP/iNK stages and iNK/mNK stages. NK cell precursors were gated on single cells, viable cells, lineage negative and defined as follows: MPP (Sca1^+^, CD244^-^, CD135^+^, CD127^-^); CLP (Sca1^-^, CD244^+^, CD135^+^, CD127^+^); preNKP (Sca1^-^, CD244^+^, CD135^-^, CD127^+^, CD122^-^); rNKP/iNK (Sca1^-^, CD244^+^, CD135^-^, CD127^+^, CD122^+^); iNK (CD244^+^, CD122^+^, NKp46^-^, CD49b^+^); mNK (CD244^+^, CD122^+^, NKp46^+^, CD49b^+^). Dotplots for both wild-type and Fra-2 transgenic mice are shown, with proportion of NK cell precursors from the parent population.

Supplementary Figure 7 Transcription factors expression level on NK precursors in the bone marrow of WT and Fra-2 TG mice. **(A)** Quantification of shown transcription factors using flow cytometry, MFI mean fluorescent intensity, lines indicate median. Statistical differences were analysed *via* mixed models using mouse genotype and cell type as fixed factors and the individual mouse as a random factor. No significant differences were observed. **(B)** Histograms of transcription factor expression on NK precursors and control stainings.

Supplementary Figure 8 Fra2 overexpression decreases ILC-1 and NK abundance. **(A, B)** Flow cytometry analysis of ILC-1 and NK cells in lung and liver tissue of WT and Fra-2 TG mice. **(A)** Gating strategy for identification of rare lymphoid cell populations. Dotplots for both wild-type and Fra-2 transgenic mice are shown, with cell proportions from the parent population. **(B)** Quantification of these populations. n=8, statistical differences were determined with a Wilcoxon Rank Sum test, **p<0.01, ***p<0.001.
