# Supplementary figures and images for: Fra-2 Is a Dominant Negative Regulator of Natural Killer Cell Development
Source: Front Immunol. 2022 Jun 22;13:909270. doi: 10.3389/fimmu.2022.909270 (PMC9257261; doi:10.3389/fimmu.2022.909270)

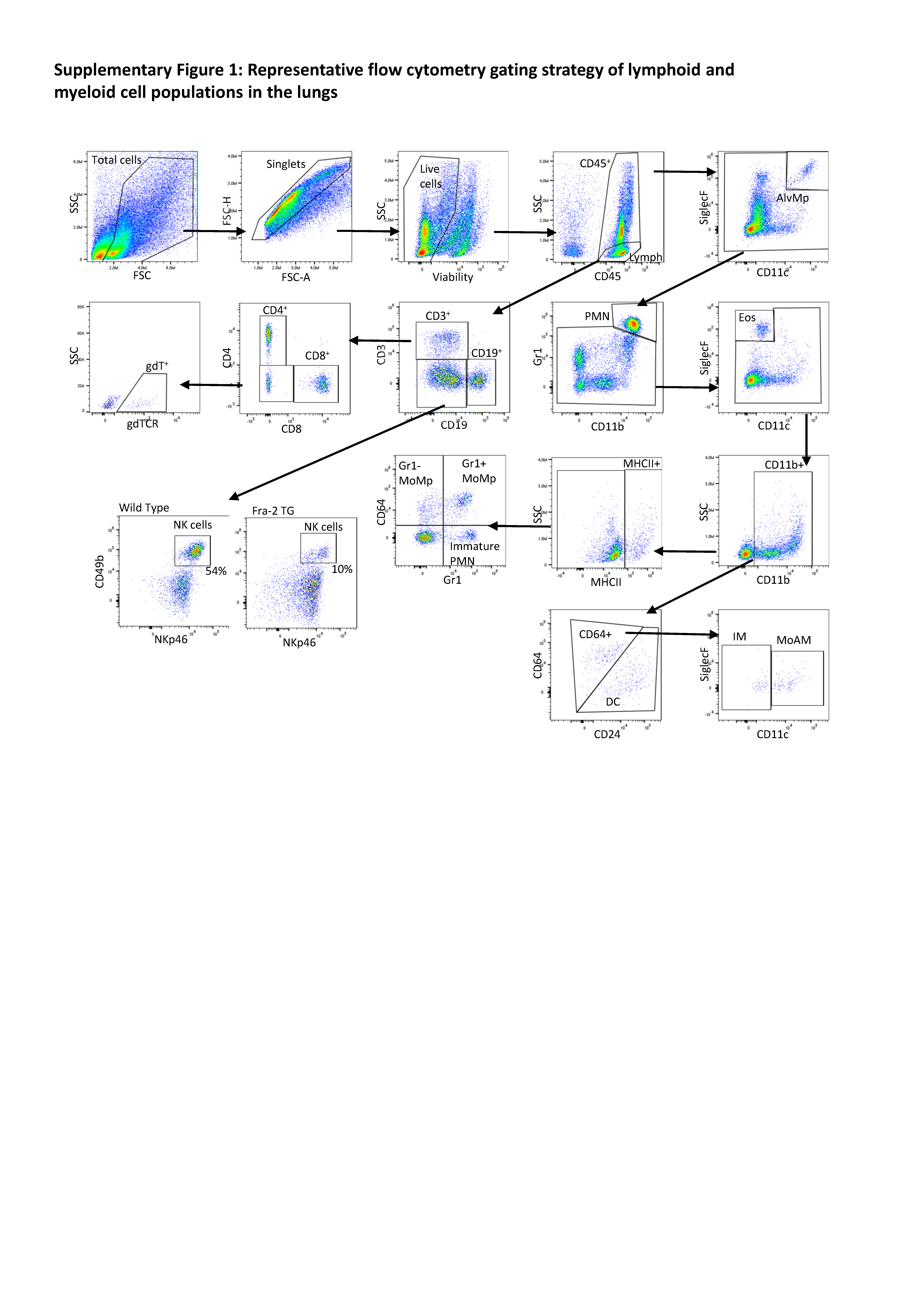

Supplement: Supplementary Figure 1 — Representative flow cytometry gating strategy of lymphoid and myeloid cell populations in the lungs. Lymphoid cells were gated on CD45+, side scatter low and B cells (CD19+), T cells (CD3+ followed by CD4/CD8/gdTCR) and NK cells (NKp46+ CD49b+). Myeloid cells were gated from CD45+ and further defined as alveolar macrophages (CD11c+, SiglecF+), neutrophils/PMN (CD11b+ Gr1+), Eos (SiglecF+), Monocytes (CD11b+, MCHII+, different populations based on their expression of CD64 and Gr1), Dendritic cells (CD11b+, MHCII+, CD24+), interstitial macrophages (CD11b+, MHCII+, CD64+, CD11c-), monocytes-macrophages (CD11b+, MHCII+, CD64+, CD11c+). Dotplots for both wild-type and Fra-2 transgenic mice are shown, with proportion of NK cells from the CD3-CD19- gate. [file Image_1.tiff]

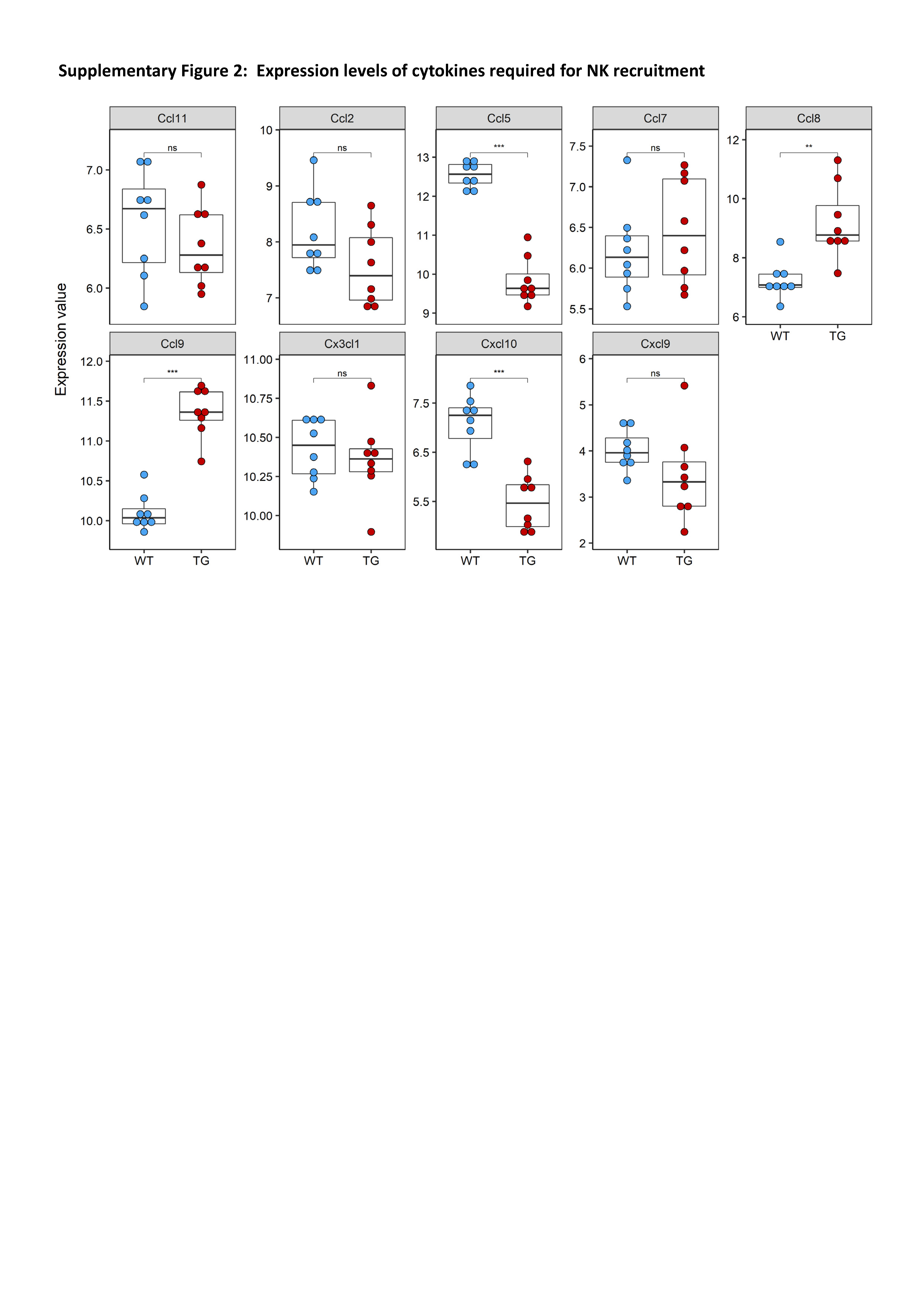

Supplement: Supplementary Figure 2 — Expression levels of cytokines required for NK recruitment. Extracted cytokine expression values in the lungs of WT and Fra-2 TG mice using DNA microarrays. Statistical differences were determined with a Wilcoxon Rank Sum test, nsp>0.05, **p<0.01, ***p<0.001, n = 8. [file Image_2.tiff]

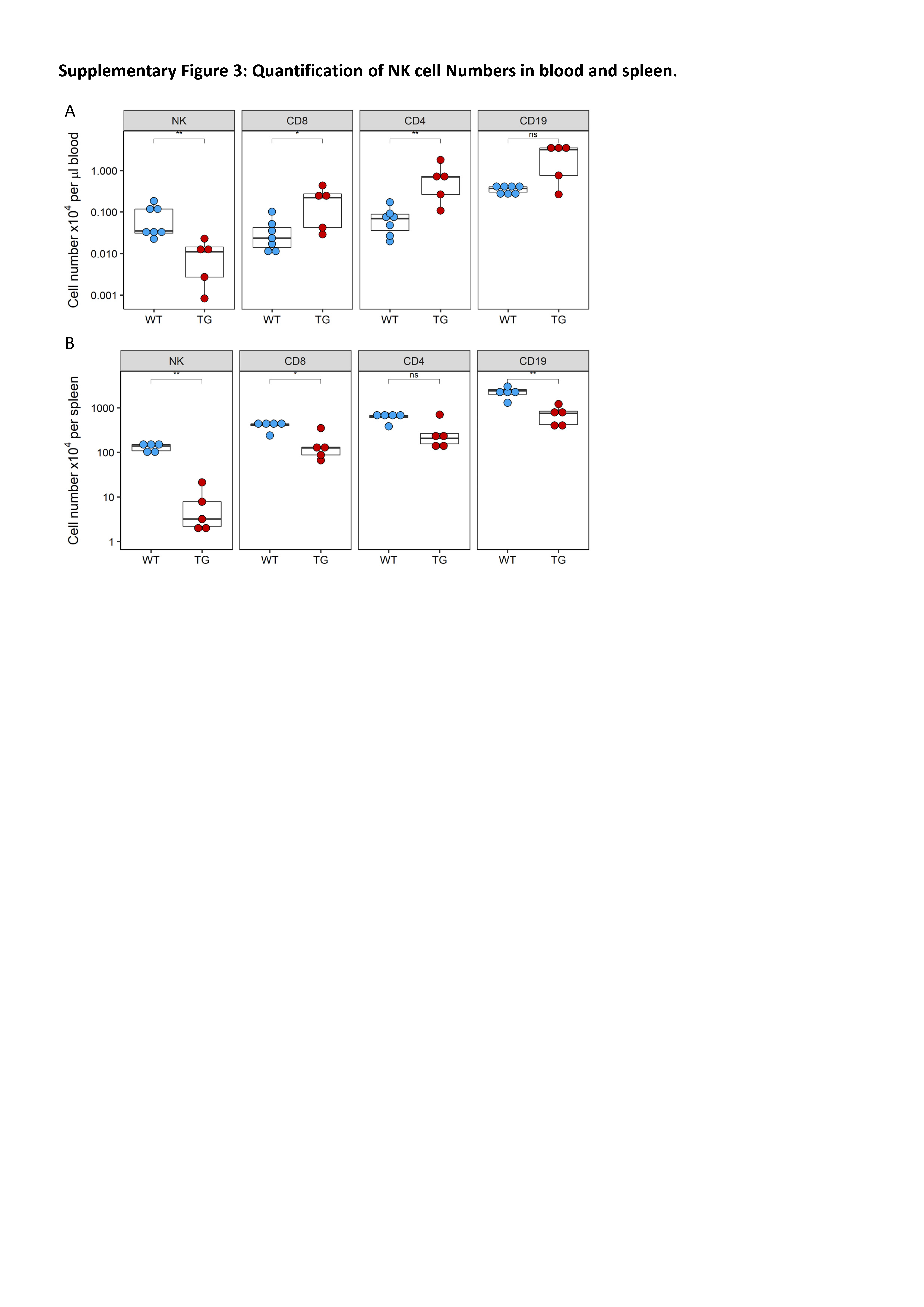

Supplement: Supplementary Figure 3 — Quantification of NK cell Numbers in blood and spleen. Absolute numbers of NK cells in the blood (A) and spleen (B) as determined by flow cytometry. Statistical differences were determined with a Wilcoxon Rank Sum test, nsp>0.05, *p<0.05, **p<0.01, n = 5-7. [file Image_3.tiff]

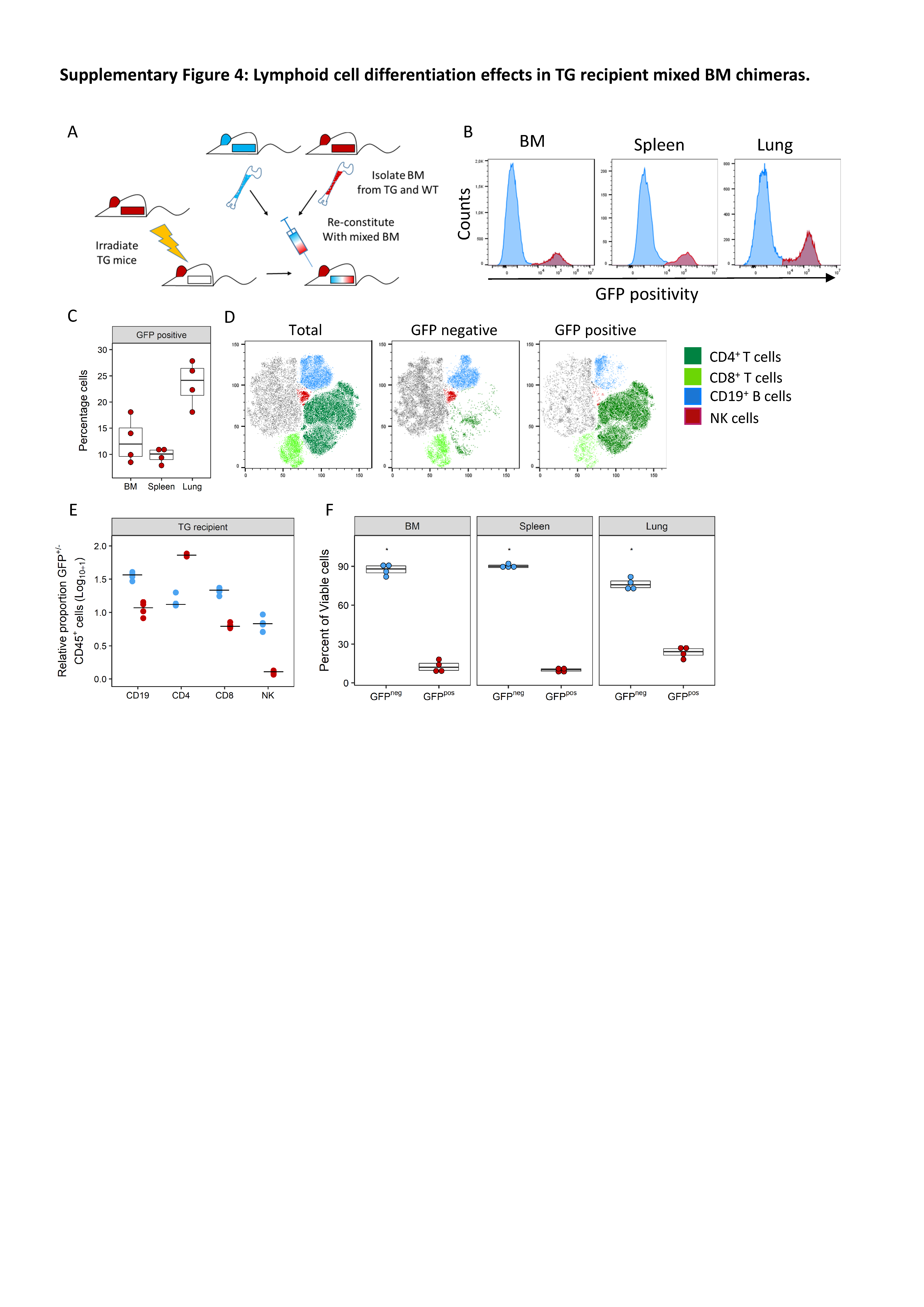

Supplement: Supplementary Figure 4 — Lymphoid cell differentiation effects in TG recipient mixed BM chimeras. (A) Schematic representation of mixed bone marrow (BM) chimera experiment. BM was extracted from WT and Fra-2 TG mice, mixed 1:1 and injected into irradiated Fra-2 TG mice. Mice were analysed six weeks post transfer. (B) Histograms of the GFP signal in total immune cells in different compartments of Fra-2 TG mice post BM reconstitution; y-axis represents percentage viable counts in the bone marrow (BM), and percentage CD45+ counts in the spleen and lung. (C) Quantification of GFP positive cells as shown in (B). (D) tSNE plots of concatenated CD45+ cells in the lung and overlaid lymphocyte populations split according to GFP positivity (negative = cells of WT origin and positive = TG origin). (E) Relative proportion of CD45+/GFP+ and CD45+/GFP- cells in the lungs of TG recipient mice. Data was log(log10+1) transformed to allow all cells to presented on the same axis. (F) Quantification of NK cell GFP positivity in the BM, spleen and lungs. Statistical differences in F were determined with a Wilcoxon Rank Sum test, *p<0.05. [file Image_4.tiff]

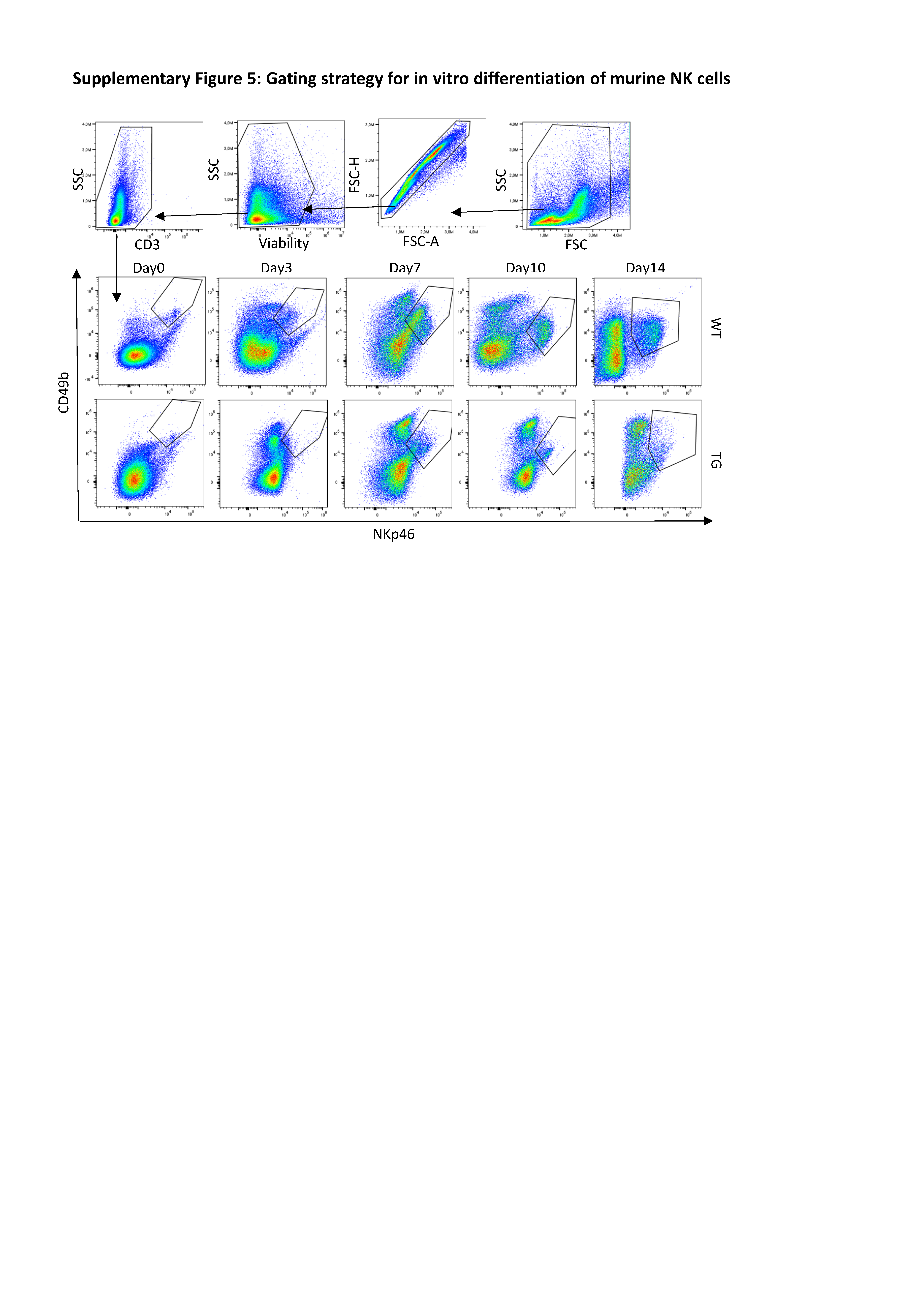

Supplement: Supplementary Figure 5 — Gating strategy for in vitro differentiation of murine NK cells. NK precursors were enriched by magnetic depletion of lineage positive cells (CD3, B220, Gr1, Ter119, CD11c and CD49b positive cells) and stimulated with FLT3L, IL-7 and IL-15 to stimulate NK cell differentiation. NK cells were defined as CD3-, CD49b+, NKp46+ cells. [file Image_5.tiff]

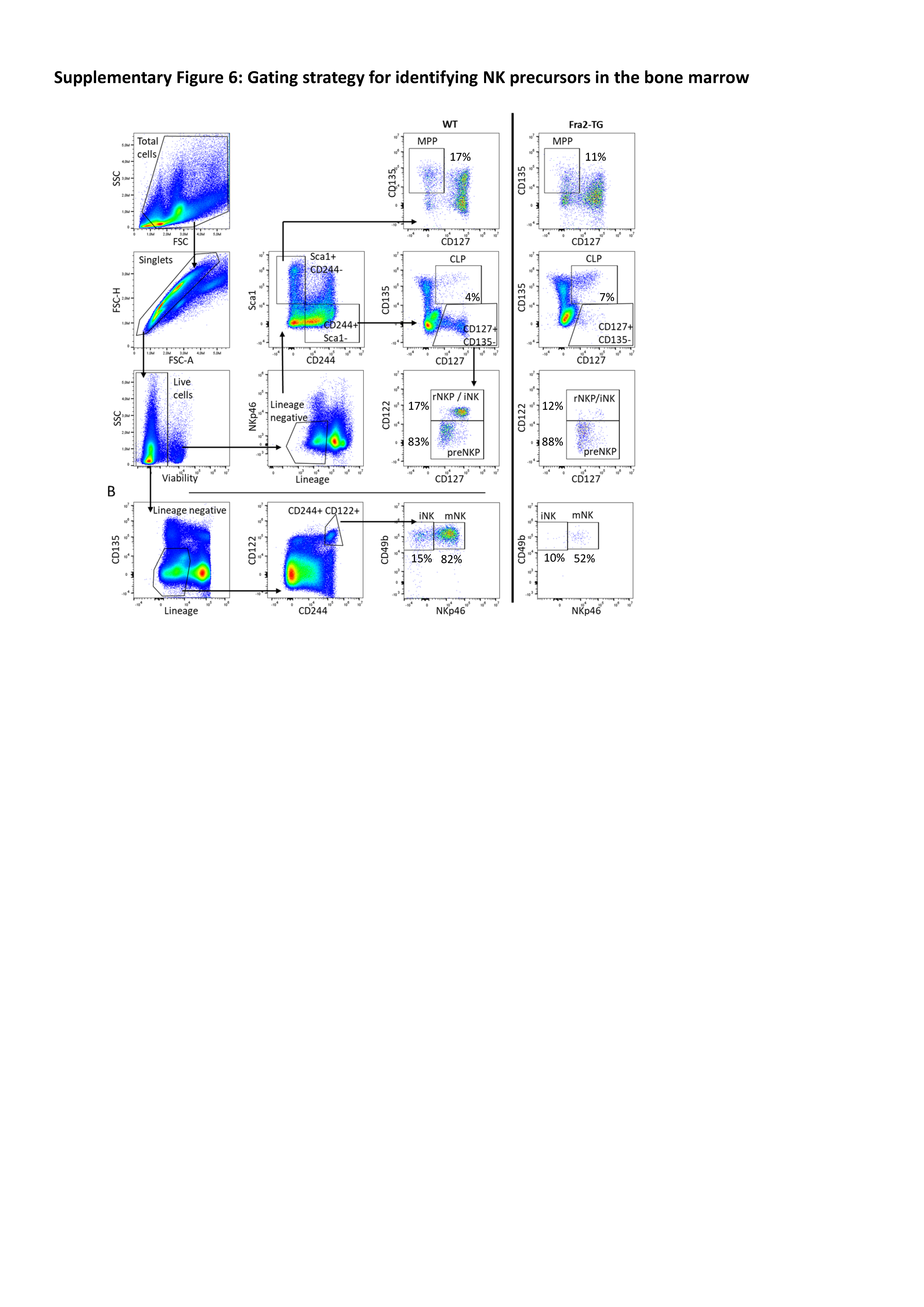

Supplement: Supplementary Figure 6 — Gating strategy for identifying and analysing NK cell precursors in the bone marrow. Bone marrow were analysed by flow cytometry using two antibody panels identify cells of the MPP- rNKP/iNK stages and iNK/mNK stages. NK cell precursors were gated on single cells, viable cells, lineage negative and defined as follows: MPP (Sca1+, CD244-, CD135+, CD127-); CLP (Sca1-, CD244+, CD135+, CD127+); preNKP (Sca1-, CD244+, CD135-, CD127+, CD122-); rNKP/iNK (Sca1-, CD244+, CD135-, CD127+, CD122+); iNK (CD244+, CD122+, NKp46-, CD49b+); mNK (CD244+, CD122+, NKp46+, CD49b+). Dotplots for both wild-type and Fra-2 transgenic mice are shown, with proportion of NK cell precursors from the parent population. [file Image_6.png]

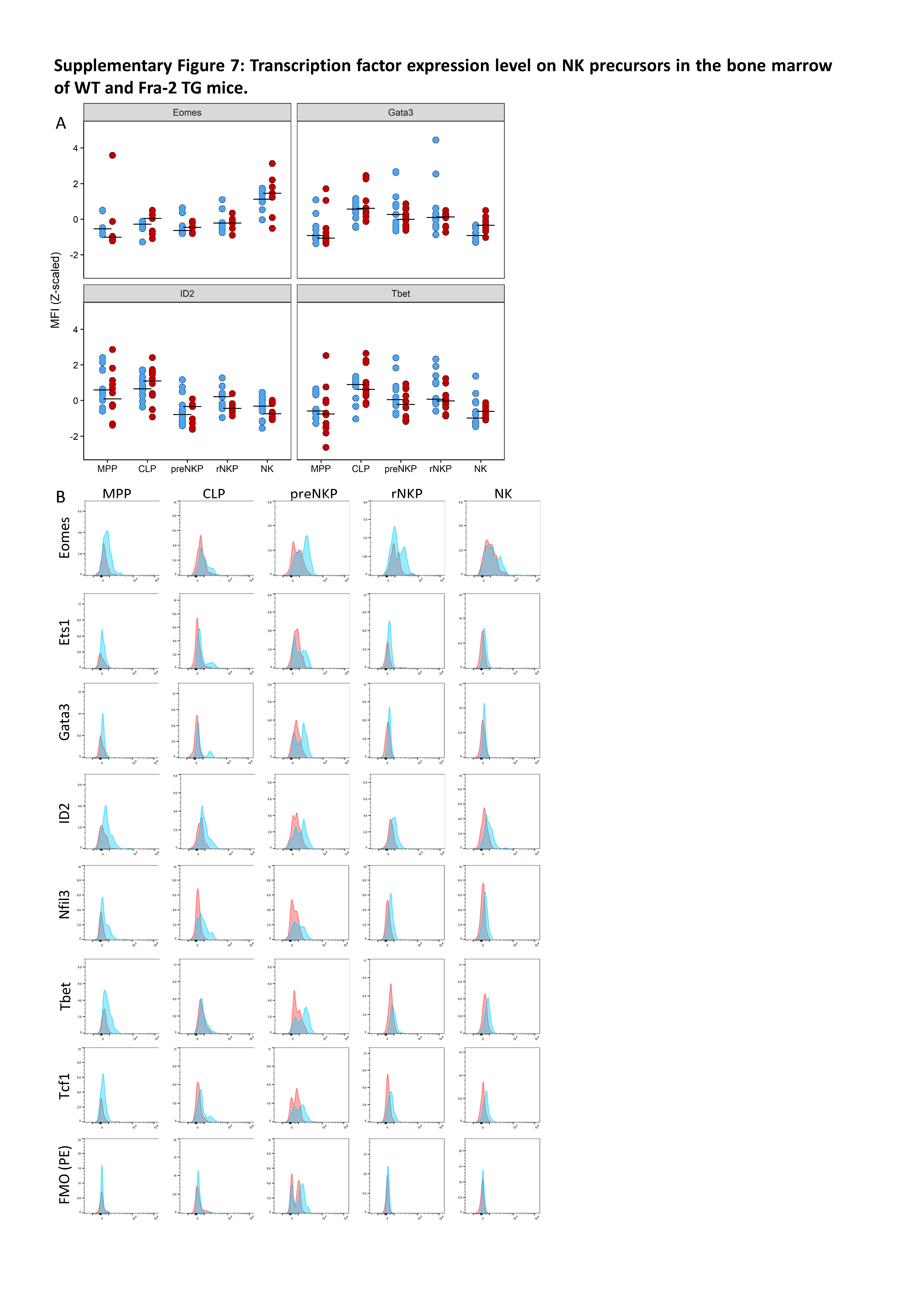

Supplement: Supplementary Figure 7 — Transcription factors expression level on NK precursors in the bone marrow of WT and Fra-2 TG mice. (A) Quantification of shown transcription factors using flow cytometry, MFI mean fluorescent intensity, lines indicate median. Statistical differences were analysed via mixed models using mouse genotype and cell type as fixed factors and the individual mouse as a random factor. No significant differences were observed. (B) Histograms of transcription factor expression on NK precursors and control stainings. [file Image_7.tiff]

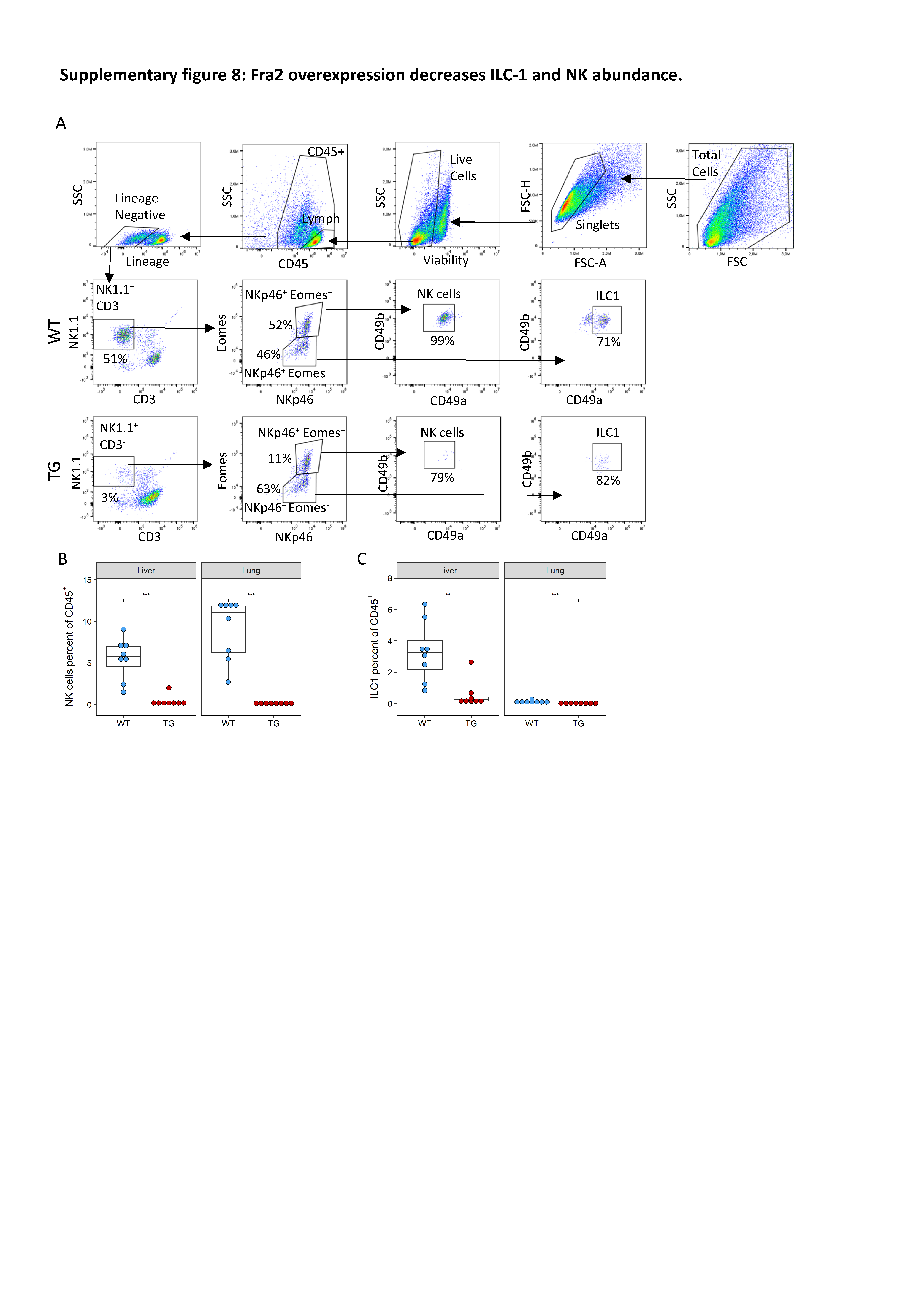

Supplement: Supplementary Figure 8 — Fra2 overexpression decreases ILC-1 and NK abundance. (A, B) Flow cytometry analysis of ILC-1 and NK cells in lung and liver tissue of WT and Fra-2 TG mice. (A) Gating strategy for identification of rare lymphoid cell populations. Dotplots for both wild-type and Fra-2 transgenic mice are shown, with cell proportions from the parent population. (B) Quantification of these populations. n=8, statistical differences were determined with a Wilcoxon Rank Sum test, **p<0.01, ***p<0.001. [file Image_8.tiff]
